# Intercomparison of Vertical Structure of Storms Revealed by Ground-Based (NMQ) and Spaceborne Radars (CloudSat-CPR and TRMM-PR)

**DOI:** 10.1155/2013/270726

**Published:** 2013-12-29

**Authors:** Veronica M. Fall, Qing Cao, Yang Hong

**Affiliations:** ^1^National Weather Center Research Experiences for Undergraduates Program, Center for Analysis and Prediction of Storms, The University of Oklahoma, National Weather Center, 120 David L. Boren Boulevard, Suite 2500, Norman, OK 73072-7309, USA; ^2^Department of Geography and Meteorology, Valparaiso University, Valparaiso, IN 46383, USA; ^3^Advanced Radar Research Center, University of Oklahoma, Norman, OK 73072, USA; ^4^Hydrometeorology and Remote Sensing Laboratory, National Weather Center, Norman, OK 73072, USA; ^5^School of Civil Engineering and Environmental Sciences, University of Oklahoma, Norman, OK 73072, USA

## Abstract

Spaceborne radars provide great opportunities to investigate the vertical structure of clouds and precipitation. Two typical spaceborne radars for such a study are the W-band Cloud Profiling Radar (CPR) and Ku-band Precipitation Radar (PR), which are onboard NASA's CloudSat and TRMM satellites, respectively. Compared to S-band ground-based radars, they have distinct scattering characteristics for different hydrometeors in clouds and precipitation. The combination of spaceborne and ground-based radar observations can help in the identification of hydrometeors and improve the radar-based quantitative precipitation estimation (QPE). This study analyzes the vertical structure of the 18 January, 2009 storm using data from the CloudSat CPR, TRMM PR, and a NEXRAD-based National Mosaic and Multisensor QPE (NMQ) system. Microphysics above, within, and below the melting layer are studied through an intercomparison of multifrequency measurements. Hydrometeors' type and their radar scattering characteristics are analyzed. Additionally, the study of the vertical profile of reflectivity (VPR) reveals the brightband properties in the cold-season precipitation and its effect on the radar-based QPE. In all, the joint analysis of spaceborne and ground-based radar data increases the understanding of the vertical structure of storm systems and provides a good insight into the microphysical modeling for weather forecasts.

## 1. Introduction

There is not much information available to diagnose large-scale vertical cloud structure and associated precipitation characteristics other than within the vicinity of ground-based radars. Spaceborne satellites and their onboard radars provide the opportunity to observe and analyze the entire vertical cloud structure. One such satellite, CloudSat, will fill this void of the ground-based radar network, expanding the data available to better understand weather systems and to produce more accurate weather models.

To diagnose storm structure, two spaceborne satellites and their radars, the CloudSat's Cloud Profiling Radar (CPR) and the TRMM's Precipitation Radar (PR), will be used. To offer cross-verification of the observations made by these two radars, a comparison with the National Mosaic and Multisensor QPE (NMQ) system, which employs the NEXRAD radar network, will be used. The vertical structure of a winter storm system will be analyzed, similar to the work done by Matrosov [1] with a west coast wintertime precipitating system. The storm chosen to be analyzed for this research occurred on the east coast on 18 January, 2009.

On 17 January, 2009, the researched storm had associated light freezing rain over Tennessee at 2000 UTC, leading to a crash involving 55 cars and trucks with over a dozen injuries, including one woman who broke both of her arms. On 18 January from 2000 to 2200 UTC, North Carolina experienced heavy snow from the same storm, with totals approaching six inches in some locations. Neither the TRMM or CloudSat satellites passed over the storm at these exact times or locations, due to CloudSat's limited spatial coverage and TRMM's latitudinal bounds. This storm, however, was still significant enough to warrant looking further into the storm structure and products through a comparison of CloudSat and NMQ as well as TRMM and NMQ.

The purpose of this paper is to use spaceborne satellites that are sensitive to precipitation and cloud hydrometeors to diagnose vertical storm structure. To cross-verify the observations made by these satellites, a comparison with a ground-based radar system (NMQ) will be performed. Specifically, the brightband level and freezing level will be compared, along with reflectivity values as measured by the different systems.

Section 2 will describe the background information on the different radar systems and satellites used in the study.  Section 3 will compare CloudSat and NMQ observations and TRMM and NMQ observations. Concluding thoughts are presented in Section 4.

## 2. Background

### 2.1. NMQ System

The National Mosaic and Multisensor Quantitative Precipitation Estimation (QPE) (NMQ) system (http://nmq.ou.edu/) is a joint project between the National Severe Storms Laboratory (NSSL), Federal Aviation Administration (FAA), National Weather Service/Office of Hydrological Development (NWS/OHD), the Office of Climate, Water and Weather Services (OCWSS), and the University of Oklahoma Cooperative Institute in Mesoscale Meteorological Studies (OU CIMMS) [2]. The project started in June 2006, along with the next generation QPE (Q2) as a means of incorporating several weather products, including radar, satellite, rain gauge, surface observations, and numerical weather prediction (NWP) to produce an all-inclusive high-resolution precipitation estimation system. The NMQ system is used internationally as a testbed for the incorporation of a 3D mosaic of multiple radars to aid in aviation and modeling, with practical applications to monitoring and warning for flooding events. Operating at high temporal and spatial resolutions of five minutes and one kilometer makes the NMQ system ideal to use in comparison with various spaceborne radars.

The NMQ system, through the collection of all the different weather products, offers a comprehensive view of storm structure. The system offers several products through which storm structure can be diagnosed, including 2D and 3D reflectivity, vertical profile of reflectivity (VPR), precipitation phase and type, and rainfall rates and rainfall totals. There is much more available through this comprehensive system, such as rain rates, rainfall totals, and vertically integrated liquid, but only a few products will be utilized for this research.

### 2.2. CloudSat CPR

The National Aeronautics and Space Administration's (NASA) Earth Science Enterprise (ESE), through various satellites, provides information about the influence clouds have on the atmosphere, including weather and climate. The afternoon “A-Train” satellite constellation is a satellite flying formation comprised of several different missions that simultaneously increase the information available about the condition of the Earth's atmosphere [3]. This constellation contains two Earth Observing System (EOS) missions and three Earth System Science Pathfinder (ESSP) missions, as well as a French Centre National d'Etudes Spatiales (CNES) mission. The five satellites fly in succession in a sun-synchronous orbit over the same locations so that the last satellite, Aura, trails the leading satellite, Aqua, by less than ten minutes. When all data from the A-Train is combined, the satellites provide synergistic information on the Earth's climate, giving a complete and thorough view of the Earth's atmosphere.

The CloudSat satellite is one of the five satellites in the A-Train constellation, located at the second flying position behind Aqua. CloudSat was launched into the A-Train formation on 28 April, 2006 and is managed and maintained by NASA's Jet Propulsion Laboratory (JPL) in Pasadena, CA. The onboard W-band nadir-pointing Cloud Profiling Radar (CPR) has been operating since 2 June, 2006, having lost only ten hours of data since the beginning of operations [4]. CloudSat operates at a high frequency of 94 GHz and short wavelength of 3 millimeters, nearly ten times more sensitive than a typical US Weather Surveillance Radar-1988 Doppler (WSR-88D). CloudSat does not have a horizontal resolution since the path width is small but has a cross-track resolution of 1.4 km and 1.8 km along-track resolution, with a vertical resolution of 500 m. The satellite is extremely sensitive at such a high frequency, with a minimum detectable signal (MDS) of <−26 dBZ, providing the opportunity for a never-before-seen perspective and analysis of vertical cloud structure, cloud composition, and cloud microphysics, even during a light precipitation event.

CloudSat offers many different products to better understand clouds' effect on the climate and cloud structure. The CloudSat2B Cloud Geometrical Profile algorithm (2B-GEOPROF) provides CPR reflectivity values, allowing for a vertical time-height cross-section of reflectivities along the CloudSat track to be generated. The Cloud Classification algorithm (2B-CLDCLASS) is another useful product and provides the cloud classification for all vertical and horizontal levels within the observed storm, with nine classifications ranging from cirrus clouds to stratus clouds to deep convection. The European Centre for Medium-Range Weather Forecast (ECMWF) model data is contained within the ECMWF-AUX files and can be accessed to create temperature profiles, which can be useful in the identification of the freezing level. Additional products include those which contain data about ice and liquid water content, rainfall rates, heating rates, and heating fluxes. All algorithms and data products are described in full detail and retrievable from the CloudSat Data Processing Center (DPC) in one granule, or one orbit, increments (http://www.cloudsat.cira.colostate.edu/).

### 2.3. TRMM PR

The Tropical Rainfall Measuring mission (TRMM) satellite's goal is to provide detailed information on the distribution of precipitation over the tropics to better understand the connection and interaction between oceans, land masses, and air masses and the shared effect on global rainfall and climate (http://trmm.gsfc.nasa.gov/). The observations made from the analysis of TRMM data have led to a better understanding of the precipitation processes that govern the tropics; therefore, more accurate models have been developed to depict these processes. The TRMM satellite was launched into orbit on 27 November, 1997 from the Tanegashima Space Center in Japan as a joint project between NASA's Goddard Space Flight Center and the Japan Aerospace Exploration Agency (JAXA). The instruments abroad the TRMM satellite include the Precipitation Radar, TRMM Microwave Imager, Visible and Infrared Scanner, Cloud and Earth Radiant Energy Sensor, and Lightning Imaging Sensor. Valuable information about the tropics is now available thanks to the information gathered from each instrument.

From the TRMM satellite, the 2A25 product used in Version 7, the TRMM Precipitation Radar (PR) Rainfall Rate and Profile Product, was used. The PR was built by JAXA in a joint contribution with the US/Japan TRMM. The PR began operating on 8 December, 1997. The PR was the first spaceborne instrument designed to provide a three-dimensional view of storm structure, providing some similar data products as those of CloudSat. The radar operates at a frequency of 13.8 GHz in the Ku-band and a wavelength of 2 cm, offering a geographic coverage from 38°S–38°N and 180°W–180°E, with a temporal resolution of sixteen orbits per day (~91.5 minutes per orbit), a horizontal resolution of 5.0 km, and vertical resolution of 0.25 km along the slant of the ray. TRMM has a swath width of 247 km with 49 rays per scan, with one scan lasting 0.6 seconds. The PR has a sensitivity less than that of CPR and is affected by Mie scattering. The PR, through all of its products, has expanded the knowledge available to understand the precipitation characteristics of the tropics.

One of the data products provided by the TRMM PR is the corrected *Z*-factor parameter, which contains a value for the attenuation corrected reflectivity factor (*Z*) at each vertical resolution level for each grid point within the TRMM swath. The reflectivity factor ranges from 0.0 to 80.0 dBZ and stores negative values of reflectivity for bad data points or ground clutter. Although there is no data available to construct a temperature profile using the PR, the product “height of freezing level” can be used to identify the 0°C isotherm. Additional data products available can distinguish rain rate, precipitation type, and precipitable water. More information about individual products or algorithms can be located on the main website for TRMM (http://trmm.gsfc.nasa.gov/overview_dir/pr.html).

## 3. Data Analysis

### 3.1. CloudSat CPR and NMQ

**Figure 1:**
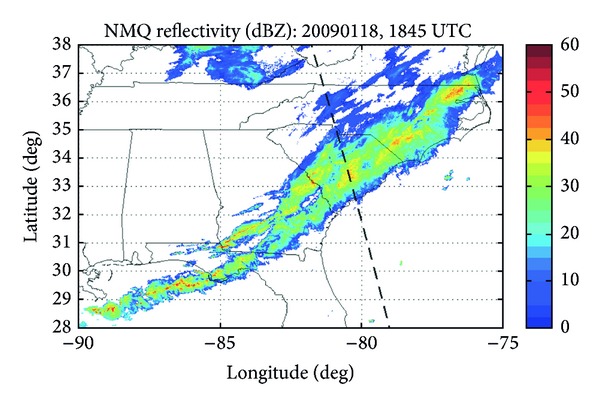
The CloudSat track has been overlaid with the NMQ composite reflectivity from 18 January, 2009 at 1845 UTC.

On 18 January, 2009, the CloudSat satellite passed over a winter storm in South Carolina at 1847 UTC, with its path dividing the state into nearly equal eastern and western halves, passing through Charleston county in the south and advancing northward through Lancaster county, covering latitude and longitude bounds of approximately 32°–35°N and 80°–82°W (Figure 1). As mentioned in Section 1, this storm had associated injuries and locally large snowfall totals.

**Figure 2:**
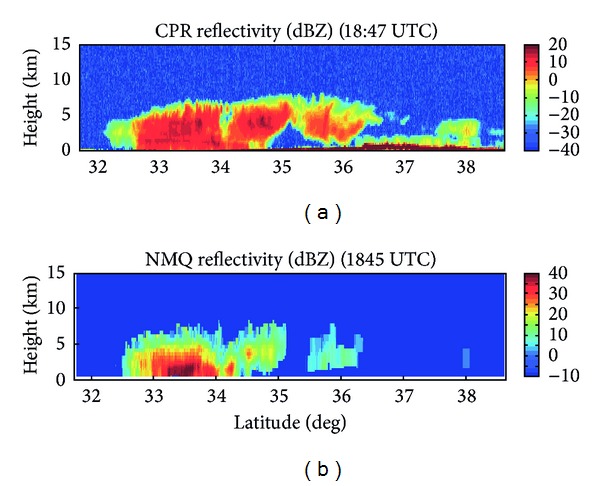
Reflectivity cross-sections from (a) CloudSat CPR and (b) NMQ.

Using the CloudSat products mentioned in Section 2.2, the storm structure and characteristics can be determined, while also using the NMQ data available to cross-validate CloudSat's observations.  Figure 2(a) shows the time-height cross-section of the CPR reflectivity through the storm. The cloud top heights from 33°–35°N are nearly constant at about 7.5 km above mean sea level (MSL) though closer to 6 km MSL at lower latitudes. Since the NMQ system is not as sensitive as CloudSat, it does not detect all of the cloud structure with small cloud particles that CloudSat has seen. In Figure 2(b), the NMQ system shows the storm only extending from 32.5°–35°N with cloud top heights of approximately 7.5 km. NMQ has captured the largest section of the storm but missed some of the finer details that CloudSat was able to detect.

**Figure 3:**
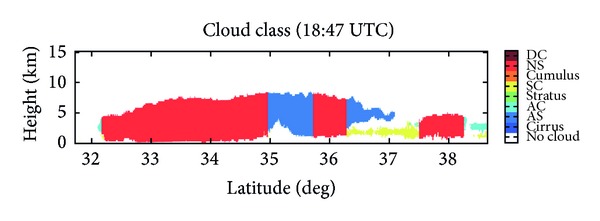
The cloud classification determined from CloudSat CPR.

Brightband features are faintly present at about 2 km MSL from about 33°-34°N on the CPR cross-section, where a tight vertical reflectivity gradient within the storm is observed, with local reflectivity maxima values of approximately 15 dBZ. The NMQ cross-section shows the largest reflectivity values in roughly the same area as CloudSat. Brightbands occur in stratiform precipitation, where a transition between hydrometeor phase from solid to liquid occurs [5]. As shown in Figure 3, the CloudSat 2B-CLDCLASS product has identified 33°–35°N as being composed of nimbostratus clouds, a form of stratiform precipitation. As ice or snow above the brightband layer begins to melt, there is a marked increase in reflectivity, as the solid hydrometeor becomes covered with a liquid water layer. This liquid casing leads to an enhanced reflectivity signal. Below the brightband layer, a large decrease in reflectivity is noted as all precipitation becomes liquid as a raindrop and the solid hydrometeor completely melts. The presence of a brightband can lead to an overestimation of precipitation rates. Using an appropriate *Z*-*R* relationship for stratiform precipitation can aid in eliminating this overestimation of rainfall rate, *R*. Identifying the location of a brightband is crucial to understanding where QPE may be incorrect. Knowing the height at which the brightband occurs is one of the advantages offered by CloudSat that will lead to better NWP.

**Figure 4:**
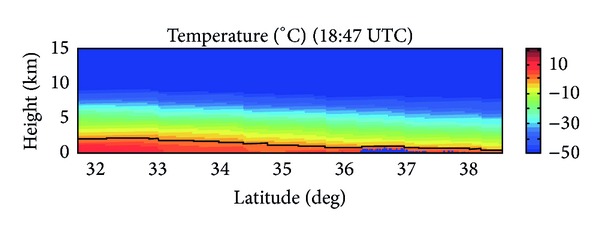
The temperature profile determined from CloudSat CPR. The dark line shows the freezing level.

The brightband layer of the CPR reflectivity cross-section and freezing level and the height of the 0°C isotherm on the CPR temperature profile are located at nearly the same height. As shown in the temperature profile of Figure 4, the freezing level at 33°N is near 2 km MSL and decreases with increasing latitude to approximately 1.5 km MSL at 35°N. Since the brightband is associated with a melting hydrometeor, it is no coincidence that the freezing level and the brightband occur at approximately the same height.

**Figure 5:**
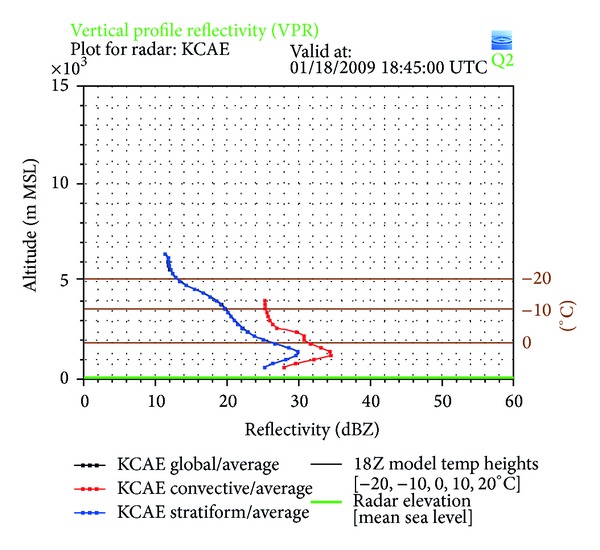
The vertical profile of reflectivity from Columbia, SC. The blue line indicates the reflectivity values for stratiform precipitation, where the peak shows the brightband level.

**Figure 6:**
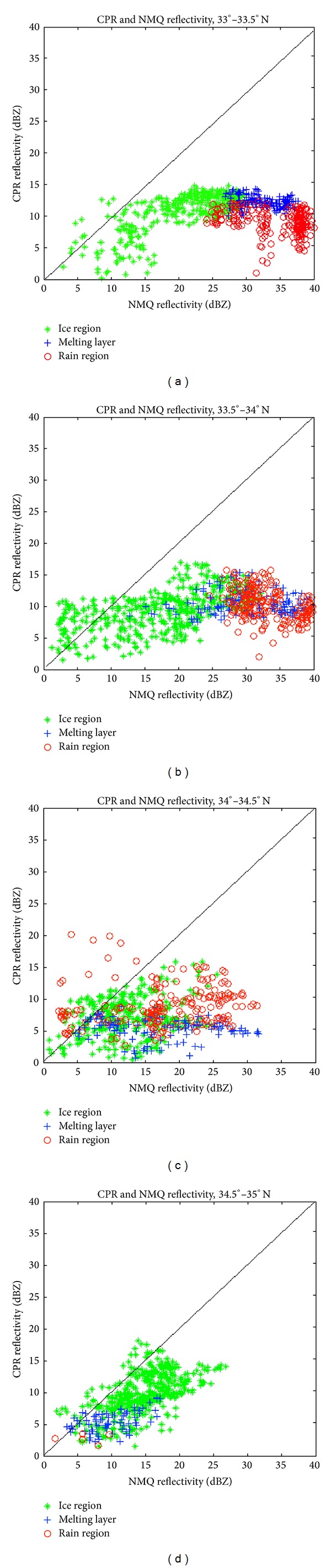
Scatterplots of NMQ reflectivity values and CPR reflectivity values from (a) 33°–33.5°N, (b) 33.5°–34°N, (c) 34°–34.5°N, and (d) 34.5°–35°N. The diamonds indicate the ice region, the crosses indicate the melting layer, and the circles indicate the rain region.

With the NMQ system, a VPR from Columbia, SC, can be used to compare the freezing level with that determined from CloudSat (Figure 5). Columbia is located at 34.0°N and 81.0°W, while CloudSat passed over the storm at 34.0°N and 80.6°W. The VPR at this specific location shows the freezing level at 1.8-1.9 km MSL while the temperature profile from the CloudSat ECMWF-AUX product shows a freezing level of approximately 2.0 km MSL. The VPR also shows the maximum reflectivity, or brightband region, for stratiform precipitation at 1.2–1.4 km MSL, while the time-height cross-section of CPR reflectivity shows the brightband around 1.8 km MSL. The VPR shows maximum reflectivity values in the brightband region of 30 dBZ, while the CloudSat cross-section records values of only 15 dBZ.  Figure 6 shows that CloudSat consistently records lower values of reflectivity when compared to those generated by NMQ and the ground-based radar network, since CloudSat operates at a higher frequency and is more sensitive to smaller hydrometeors than the NMQ system. This trend is especially evident from 33°-34°N where the brightband layer is most prevalent in the CloudSat reflectivity cross-section. As expected, where a brightband is present from 33°-34°N, *Z* has very high values of at least 25 dBZ for the NMQ data and 10 dBZ for CloudSat. From 34°-35°N, the cross-section does not show a brightband and the temperature profile shows the freezing level dropping off to less than 1.5 km MSL, so that there are not significantly large *Z* values in this region. The important trend to note is that where NMQ has recorded its largest reflectivity values, so has CloudSat, located within the melting layer at the brightband.

### 3.2. TRMM PR and NMQ

**Figure 7:**
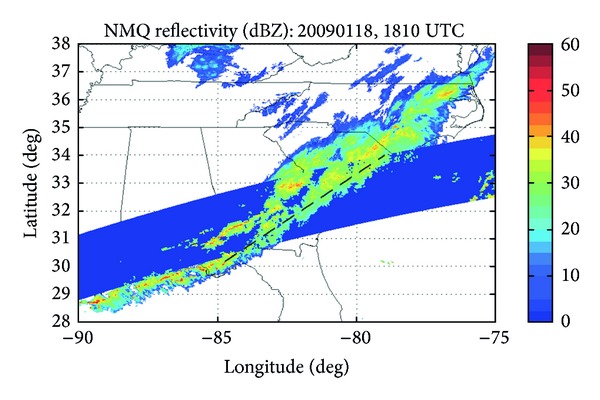
The TRMM track shown over the NMQ composite reflectivity from 18 January, 2009 at 1810 UTC.

At 1810 UTC, the TRMM satellite observed the same storm over the southeastern US about forty minutes before CloudSat. The chosen path to analyze stretches from Grady County in the far southwest of Georgia and extends northeastward to Horry County in eastern South Carolina, bounded approximately by 30°–34°N and 79°–85°W (Figure 7).

**Figure 8:**
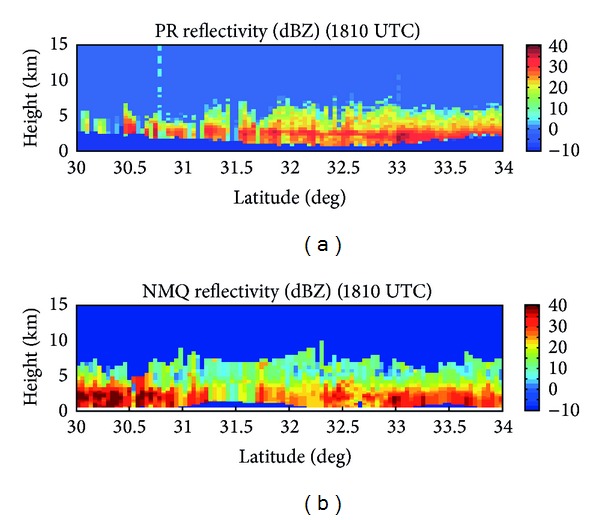
Reflectivity cross-sections from the (a) TRMM-PR and (b) NMQ.

The vertical resolution of the TRMM satellite is worse than that of CloudSat, but the same features are still distinguishable. From 30°–32°N, the cloud tops are very low, at heights less than 5 km MSL in most cases, but the cloud tops stay fairly consistent around 5 km MSL from 32°–34°N (Figure 8(a)). A distinct brightband can be found from 31.5°–33°N at 2 km MSL in the TRMM cross-section, with reflectivity values approaching 30 dBZ. The NMQ cross-section does not show as strong of a brightband within these same latitudes (Figure 8(b)). A brightband can be seen from 32.5°–33°N, but there is also a second brightband on the NMQ cross-section from 30°-31°N that does not appear on from the PR.

**Figure 9:**
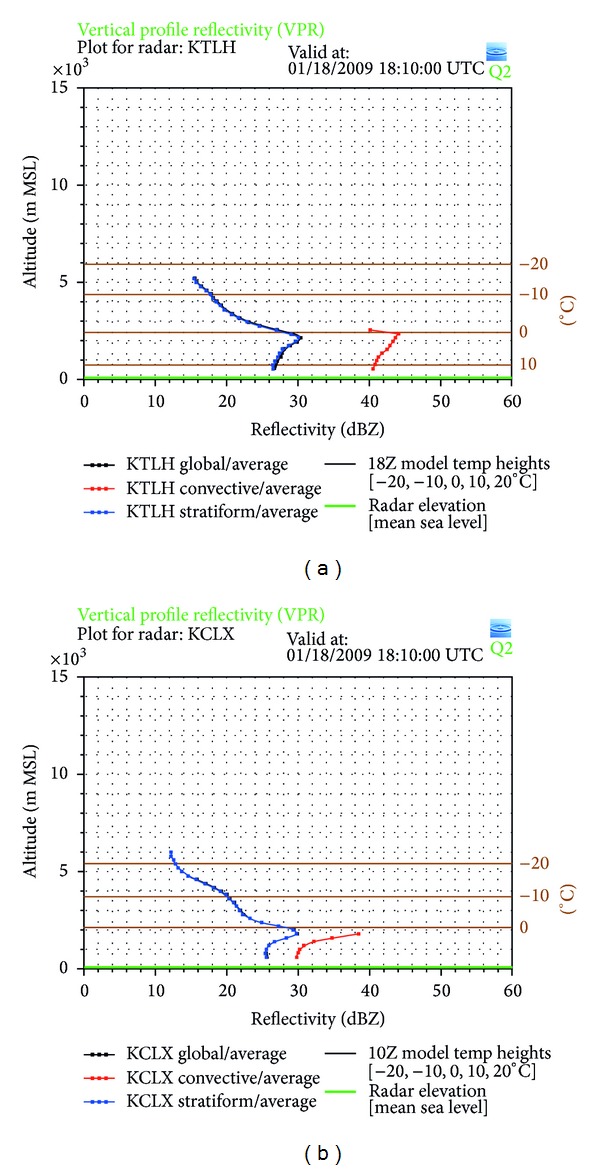
Vertical profiles of reflectivity from (a) Tallahassee, FL, and (b) Charleston, SC. The dark line shows the reflectivity values for stratiform precipitation, where the peak shows the brightband.

Since the PR was developed to study precipitation, a temperature profile is not available through this dataset. The NMQ system's VPRs, however, may still be used to compare the brightband levels as well as to see where the freezing level occurs in comparison to the brightband. The chosen TRMM track has passed closely within two different VPR locations, one in Tallahassee, FL (KTLX), and the other in Charleston, SC (KCLX) (Figure 9). Tallahassee is located at 30.5°N and 84.3°W and Charleston at 32.8°N and 79.9°W. For stratiform precipitation, these locations show the brightband and maximum values of reflectivity occurring at a height of 2.2 km MSL in Tallahassee, while the height at Charleston is 1.8 km MSL. In Tallahassee, the maximum value for reflectivity in the brightband is determined to be 30 dBZ, agreeing with the observations from the PR, while, at the Charleston location, the maximum reflectivity is also approximately 30 dBZ. The freezing level is also slightly lower in Charleston than Tallahassee, as expected, where the height is 2.5 km MSL in Tallahassee and 2.2 km MSL in Charleston. Since Charleston is north of Tallahassee, it is an expected observation to see that the height of the freezing level decreases with increasing latitude and that the brightband has exhibited the same behavior, with each height measurement changing about a third of a kilometer between the two locations, which are separated by a distance of 484 km.

**Figure 10:**
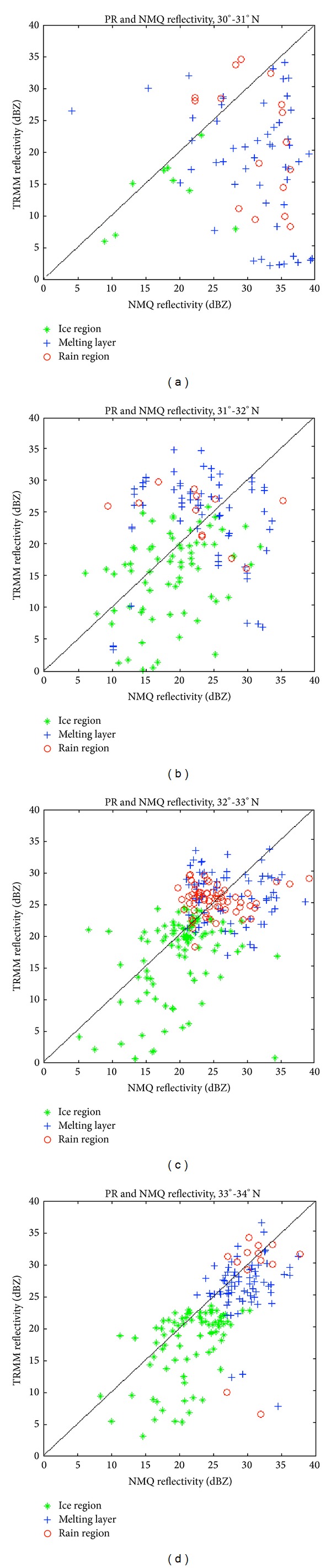
Scatterplots of NMQ reflectivity values and PR reflectivity values from (a) 30°-31°N, (b) 31°-32°N, (c) 32°-33°N, and (d) 33°-34°N. The diamonds indicate the ice region, the crosses indicate the melting layer, and the circles indicate the rain region.

Reflectivity values for the TRMM-PR and NMQ system were compared as scatterplots (Figure 10). In the location of a brightband, from 32°–34°N, the data points are not spread too far apart and lie close to the *x*-*y* line. The melting level region has recorded the largest reflectivity values for both TRMM and NMQ, as expected. In the melting layer, TRMM has recorded maximum reflectivity values of approximately 35 dBZ, while NMQ recorded maximum values of nearly 40 dBZ. Outside the main brightband region from 30°–32°N, the data points are much more scattered and not as well clustered within each of the levels analyzed. Though the trend is not as evident as the CPR and NMQ comparison, the PR has recorded slightly lower reflectivity values than NMQ, since the PR operates at a higher frequency than the WSR-88D radars used by the NMQ system. TRMM and NMQ have cross-verified observations though by showing that large reflectivity values are found within the melting layer at the location of the brightband.

## 4. Conclusion

The CloudSat satellite's Cloud Profiling Radar is very useful in the diagnosis of storm structure, due to the products that show the cross-section of reflectivity and temperature profile and also cloud classification and precipitation type. The CPR has a better resolution than the TRMM PR and is able to provide important information about the different levels within a storm, specifically above the freezing level and at the freezing level itself. To provide supplementary information, the ground-based NMQ radar system was used, which incorporates several weather products and will lead to improved quantitative precipitation estimation.

Through the combination of data available from ground and spaceborne radars, our understanding of vertical cloud structure has increased. Using different radar systems allows for cross-verification of observations, as shown by comparing the reflectivity cross-sections, temperature profile, and VPRs. This combination also allows for the classification of hydrometeors and microphysical retrieval through the usage of multimeasurements. Using CloudSat and TRMM products, the NMQ system has been used to verify the freezing level and brightband level, as well as to provide a comparison of the reflectivity values as recorded by the different systems. Through the analysis and comparison of these different radar systems, a better understanding of the vertical storm structure will lead to improved quantitative precipitation estimation and forecast modeling.
